# Quality of life of early stage colorectal cancer patients in Morocco

**DOI:** 10.1186/s12876-016-0538-9

**Published:** 2016-10-12

**Authors:** Hind Mrabti, Mounia Amziren, Ibrahim ElGhissassi, Youssef Bensouda, Narjiss Berrada, Halima Abahssain, Saber Boutayeb, Samira El Fakir, Chakib Nejjari, Abdellatif Benider, Nawfel Mellas, Omar El Mesbahi, Maria Bennani, Rachid Bekkali, Ahmed Zidouh, Hassan Errihani

**Affiliations:** 1 Department of Medical Oncology, National Institute of Oncology, University Hospital, Rabat, Morocco; 2 Department of Epidemiology and Public health, Faculty of Medicine, Fez, Morocco; 3 Department of Radiotherapy, Ibn Rochd University Hospital, Casablanca, Morocco; 4 Department of Medical Oncology, Hassan II University Hospital, Fez, Morocco; 5 Lalla Salma Foundation-Prevention and Treatment of Cancers, Rabat, Morocco

**Keywords:** Quality of life, EORTC QLQ C30, Early, Colorectal cancer

## Abstract

**Background:**

A multicentre cohort study was held in Morocco, designed to evaluate the quality of life of cancer patients. The aim of this paper is to report the assessment of the quality of life of early colorectal cancer patients, before and after cancer treatment, to identify other factors which are related to this quality of life.

**Methods:**

We used the third version of the QLQ-C30 questionnaire of the European organization for Research and treatment of Cancer (EORTC) after a transcultural validation. The Data collection was done at inclusion and then every twelve weeks to achieve one year of follow up.

**Results:**

Overall 294 patients presented with early colorectal cancer, the median age was 56 years (range: 21–88). The male–female sex ratio was 1.17. At inclusion, the global health status was the most affected functional dimension. For symptoms: financial difficulties and fatigue scores were the highest ones. Emotional and social functions were significantly worse in rectal cancer. Most symptoms were more present in rectal cancer. At inclusion, global health status score was significantly worse in stage III. Anorexia was significantly more important among colorectal female patients. For Patients over 70 years-old, the difference was statistically significant for the physical function item which was lower. Overall, Functional dimensions scores were improved after chemotherapy. The symptoms scores did not differ significantly for patients treated by radiotherapy, between inclusion and at one year.

**Conclusion:**

Our EORTC QLQ C30 scores are overall comparable to the reference values. Neither chemotherapy, nor radiotherapy worsened the quality of life at one year.

## Background

Colorectal cancer is frequent in western countries, it represents the third most common cancer in France, with approximately 40 000 new cases per year [1]. According to Moroccan registries, it represents the first gastro-intestinal cancer before gastric cancer, and the fourth most common cancer, with an incidence of 8,4/100 000 habitants in 2007 [2]. This incidence have increased these last years (it was 6.9/100 000 habitants in 2005) [2]. This could be explained by the fact that Morocco has experienced, over the past 5 years, considerable progress in the detection, diagnosis and treatment of cancer. Many anticancer centers, equipped with the latest technologies have been constructed, most of anti-cancer drugs are available, and a national cancer plan (2010–2019) has been established.

When improving treatment of cancer, quality of life (QOL) becomes an important parameter to assess. It permits the evaluation of the impact of cancer and its treatment on the overall life of patients.

A multicentre cohort study was held in Morocco, designed to evaluate the quality of life of Moroccan cancer patients, including colorectal, cervical, breast and lung cancer [3, 4]. It is a prospective multicentre cohort study, performed in the ten existing oncological centers in Morocco.

The aim of this paper is to report the assessment of the quality of life of early colorectal cancer patients, before and after specific cancer treatment (chemotherapy and/or radiotherapy), to identify other factors which are related to this quality of life (age, sex, localization: colon or rectum, performance status, disease stage), to describe Moroccan cancer patients characteristics.

## Methods

### Study design and study population

This is a Moroccan multicentre prospective cohort study. Patients were included, between December 2009 and December 2011, in the ten existing cancer centers in Morocco: seven public institutions (National Institute of oncology and Mohammed V military hospital in Rabat, Ibn rochd University hospital in Casablanca, Oujda, Agadir, Marrakech, Fez) and 3 private cancer clinics (Al Kindy and le Littoral in Casablanca, Nakhil in Rabat).

All patients were adults of 18 years or older, newly diagnosed with a histologically proven colorectal cancer. The diagnosis had to be made in less than three months before inclusion. Patients operated for their cancers within 3 months before inclusion were eligible to enter the study. A written informed consent was required for enrolment in the study, after the patient has received all the informations regarding the conditions of the study. Ethical approval was obtained from the ethics committees of the University Hospital Center Hassan II in Fez. Patients who received a specific treatment, chemotherapy or radiotherapy, and those with major neuropsychological defect were excluded.

In this paper we report quality of life assessment of early stage colorectal cancer patients which was defined by localized, stage I-II and III, colorectal cancer. Metastatic colorectal cancer (stage IV) was excluded, and will be separately analyzed, because patients are treated in a palliative setting, with systemic therapy and all patients remain under treatment at one year.

### Collection data procedure

#### Questionnaires and data collection

We used the third version of the QLQ-C30 questionnaire of the European organization for Research and treatment of Cancer (EORTC). It is a validated tool for the assessment of QoL in cancer patients. It contains 14 items: 5 of them are related to activity (Physical, professional and leisure, cognitive, emotional and social functioning), 3 of them to symptoms (Asthenia, pain, nauseas and vomiting) and the remaining six are independent items evaluating other symptoms (dyspnea, insomnia, appetite loss, constipation, diarrhea, and financial difficulties). The QLQ-C30 was used after a transcultural validation [3]. It was developed according to Beaton and al recommendations [5].

The QLQ-C30 is a self-reporting questionnaire; however it was administered to illiterate patients by trained interviewers. The Data collection was done at inclusion and then every three months to each patient, until one year of follow-up.

All socio-demographic, economic and therapeutic data were extracted for colorectal cancer : age, gender, geographic origin, marital status, profession, income, level of education, medical insurance, localization, histological subtype, Tumor-Node-Metastasis (TNM) staging, evaluation of performance status according to Eastern Cooperative Oncology Group (ECOG) criteria, treatment (surgery, pre-operative radiotherapy or concurrent chemo-radiation for rectal cancer, adjuvant chemotherapy).

### Statistical methods

- Calculation of EORTC QLQ-C30 scores [6]:

Scores of the items were linearly transformed to a scale from 0 to 100. A high score for a functional scale represents a healthy level of functioning, a high score for the global health status represents a high quality of life, but a high score for a symptom item represents a high level of symptomatology.

- Descriptive analysis

Descriptive analysis was performed for sociodemographic and clinical features, with a confidence interval defined at 95 %. To describe the quality of life scores, we calculated the means and standard deviations, minimum and maximum.

- Univariate analysis:

At inclusion, a global analysis was conducted in the whole population of early colorectal patients. Subgroup analyses were performed according to some variables: age, gender, tumor location (colon versus rectum), performance status (PS ≤ 1 versus >1) and stage. The comparison between subgroups was performed by t-tests and one-way ANOVA. A *p*-value of less than 0.05 was considered as statistically significant. To assess the scores variability of quality of life between inclusion (before treatment: chemotherapy or radiotherapy) and at 1 year (after treatment), we used the paired Student’s *t*-test. The statistical analysis was performed by SPSS, version 17.0, software.

## Results

Overall 2903 cancer patients were recruited, among whom 461 patients presented with colorectal cancer, 294 (63.7 %) were diagnosed at an early stage (I, II or III).

### Socio-demographic and clinical characteristics of early colorectal cancer patients

Two hundred ninety-four patients with an early stage colorectal cancer were included in the study, of which there were 161 colon cancers (54.7 %) and 133 rectal cancers (45.3 %). The median age at diagnosis was 56 years (range: 21–88). The male–female sex ratio was 1.17. Majority of patients came from urban areas (78.2 %), a significant number were illiterate (49 %), and only 7.5 % had a high level of education. 32.7 % of patients were professionally active, but the majority of them had a low-intermediate socio-economic status. About 60 % of patients had no medical insurance. Eighty percent were married (see Table 1).

**Table 1:** Sociodemographic characteristics of colorectal cancer patients at inclusion, Morocco, 2009–2011

	No (total 294)	%
Age (median)	56 years (range : 21–88)	
Sex		
• Male	159	54.1
• Female	135	45.9
Living environment		
• Urban	204	78.2
• Rural	57	21.8
Residency		
• Casablanca	29	10.2
• Rabat	33	11.6
• Agadir	9	3.2
• Meknes	9	3.2
• Kenitra	9	3.2
• Marrakech	35	12.3
• Others	170	57.9
Education		
• Illiterate	144	49.0
• Primary school	55	18.7
• College	73	24.8
• High school	22	7.5
Marital status		
• Single	32	10.9
• Married	235	80
• Divorced	10	3.4
• Widower	17	5.8
Professional Status		
• Working	96	32.7
• Retired	41	13.9
• Without profession	49	16.7
• Housewife	106	36.1
• Student	2	0.7
Socioeconomic Status		
• Low	142	48.3
• Intermediate	141	48.0
• High	11	3.7
Medical insurance		
• Public health-insurance	108	36.7
• Private insurance	10	3.4
• No medical insurance	176	59.9

Tumor-node-metastasis staging system was as follows: Stage I = 19 (6.5 %), stage II = 80 (27.2 %), stage III = 195 (66.3 %). One hundred and ninety eight patients (67.3 %) patients received adjuvant chemotherapy based on the combination of capecitabin and Oxaliplatin every three weeks, during 6 months (eight cycles). Among rectal cancer patients (133), 50 (37.5 %) received pre-operative radiotherapy, which was concomitant to chemotherapy in 40 patients. (See Table 2).

**Table 2:** Clinical features of early colorectal cancer patients, Morocco, 2009–2011

	No (total 294)	%
Localization		
• Colon	161	54.7
• Rectum	133	45.3
TNM Staging		
• Stage I	19	6.5
• Stage II	80	27.2
• Stage III	195	66.3
Surgery		
• Radical surgery	113	38.4
• Conservative surgery	67	22.8
• Palliative surgery	5	1.7
• Unknown	109	37.1
Radiotherapy (rectal cancer)	133	
• Performed	50	37.5
• Not performed	83	62.5
Adjuvant chemotherapy		
• Performed	198	67.3
• Not performed	77	26.2
• Unknown	19	6.5

This study have some limitations: mainly data concerning surgery are missed. The surgery have been performed, in majority of patients (223) (except those treated by pre-operative radiotherapy) before inclusion in the study. At inclusion, 50 patients presenting with rectal cancer have not been operated because they were candidate for pre-operative radiotherapy. The surgical procedure was unknown in 37 % of patients (Table 2). Therefore, the QOL have not been assessed before surgical treatment.

### QOL assessment

At inclusion, all patients were assessed before receiving any specific treatment: radiotherapy or chemotherapy.

At inclusion, the global health status was the most affected functional dimension (score 62.84). For symptoms: financial difficulties and fatigue scores were the highest ones (61.72 and 38.21 respectively). The scores at inclusion are summarized in Tables 3 and 4.

**Table 3:** Inclusion mean scores of functional dimensions according to sex, age, localization, stage and Performance status, among colorectal cancer patients, Morocco, 2009–2011

Subgroup	Global health status	Physical function	Role function	Emotional function	Cognitive function	Social function
All	62.8499	71.6667	67.5641	65.6595	86.0051	81.9338
Localization						
Colon	63.4199	70.6494	67.9825	68.7049*	86.5801	85.6061*
Rectum	62.0370	73.1308	66.9753	61.3169	85.1852	76.6975
				*p* = 0.022		*p* = 0.005
Stage						
I	66.6667	69.8148	74.0741	68.5185	91.6667	88.8889
II	68.3824	72.9657	67.1642	69.2402	89.9510	81.3725
III	60.3220*	71.3524	67.0476	63.9836	83.9015	81.4394
	*p* = 0.003					
Sex						
Male	64,0443	71,4085	69,4836	67.4825	85.5478	83.3333
Female	61,4146	71,9748	65,2542	63.4687	86.5546	80.2521
Age						
< 50	63.5294	74.5490*	71.7647	65.3595	85.2941	83.9216
50–60	63.5294	72.2603	65.9722	65.0304	89.2694	84.2466
60–69	62.9944	73.6782	70.3390	67.0904	85.3107	77.1186
≥ 70	58.8889	62.6667	58.3333	65.3704	82.9630	80.7407
		*p* = 0.033				
PS						
< 1	70.2236*	80.8333*	79.2683*	69.9864	90.8537*	92.6829*
≥ 1	59.4907	67.4674	62.1723	63.6883	83.7963	77.0370
	*p* < 0 .001	*p* < 0 .001	*p* < 0 .001		*p* < 0 .001	*p* < 0 .001

*: statistically significant

**Table 4:** Inclusion mean scores of symptoms according to sex, age, localization, stage and Performance status, among colorectal cancer patients, Morocco, 2009–2011

Subgroup	Fatigue	Nausea	Pain	Dyspnea	Insomnia	Anorexia	Constipation	Diarrhea	Financial difficulties
All	38.2103	11.3868	30.1527	15.2672	32.3116	32.3116	20.4835	18.3206	61.7188
Localization									
Colon	35.7504	8.9827*	25.3247*	13.4199	21.8615*	28.5714*	14.9351*	14.5022	62.6398
Rectum	41.7181	14.8148	37.0370	17.9012	35.1852	37.6947	28.3951	23.7654	60.4361
		*p* =0.028	*p* =0.001		*p* =0.001	*p* = 0.024	*p* < 0.001	*p* = 0.006	
Stage									
I	37.0370	15.7407*	23.1481	11.1111	25.9259	29.6296	18.5185	18.5185	61.1111*
II	35.8660	4.4118	26.7157	12.2549	22.5490	28.9216	13.7255	18.1373	52.0202
III	39.2361	13.6364	32.1970	16.8561	29.3561	33.9048	23.2955	18.3712	65.5039
		*p* = 0.006							*p* = 0.033
Sex									
Male	36.2082	11.6550	27.3893	14.6853	26.3403	28.4038*	18.1818	19.8135	61.2293
Female	40.6162	11.0644	33.4734	15.9664	36.9748	36.9748	23.2493	16.5266	62.3188
						*p* = 0.032			
Age									
< 50	36.4052	13.1373	30.3922	16.4706	30.5882	35.3175	23.1373	18.0392	65.4618
50–60	36.5297	8.9041	27.3973	11.8721	27.3973	27.3973	17.3516	20.5479	60.0939
60–69	38.4181	11.2994	31.6384	13.5593	23.1638	34.4633	16.3842	16.9492	57.4713
≥ 70	44.0741	12.2222	32.2222	20.7407	26.6667	31.8519	25.9259	17.0370	62.8788
PS									
< 1	26,8970*	6,5041*	20,5285*	11,3821*	17.0732	24.7967*	11.7886*	15.4472	65.0407
≥ 1	43,3642	13,6111	34,5370	17,0370	32.0370	35.7542	24.4444	19.6296	60.1533
	*p* < 0.001	*p* = 0.012	*p* < 0.001	*p* < 0.001		*p* = 0.01	*p* = 0.001		

*: statistically significant

### Localization specific scores

The global health status had the lowest score at inclusion for both colon and rectal cancer patients. The less affected dimension was the cognitive function (scores 86 and 85 for colon and rectal cancers respectively). Differences were statistically significant between the two localizations for emotional and social functions. They were worse in rectal cancer (61 vs 68 and 76 vs 85 respectively) (see Table 3).

Most symptoms were more present in rectal cancer. The difference was statistically significant for Nauseas, pain, insomnia, anorexia, constipation and diarrhea (See Table 4).

### Stage specific scores

At inclusion, global health status score was significantly worse in stage III (*p* = 0.003). Moreover, Role, emotional and cognitive functions were more altered in stage III colorectal cancer but the difference was not statistically significant (see Table 3). In addition to financial difficulties, the prominent symptom in all stages was the fatigue. The scores were 37, 39 and 36 for stages I, II and III respectively. Curiously, nausea was significantly higher in stage I (See Table 4).

### Sex specific scores

Overall, women had more altered functional functions and more permanent symptoms (see stables 3 and 4). Anorexia was significantly more prevalent among colorectal female patients (28 versus 37) (see Table 4).

### Age specific scores

Patients over 70 years-old had lower scores, for global health status, physical role and cognitive functions. The difference was statistically significant for the physical function item (see Table 3). Major symptoms in all age subgroups were financial difficulties (scores ranges between 57 and 65) and fatigue (scores 36 to 44). There was no significant difference between symptoms according to the age range. Nevertheless, descriptive data suggested that patients of more than 70 years-old had higher scores of pain, dyspnea and constipation. Patients between 60 and 69 years-old had more diarrheas while patients less than 50 years-old had higher scores of nauseas and insomnia (see Table 4).

### Performance status (PS) specific scores

Majority of patients had PS >1 (67.9 %) at inclusion (see Table 2). Except for the emotional function, all of the functional dimensions were significantly more altered in patients with PS > 1 (see Table 3). In the same way, most symptoms scores were higher in “PS > 1” patients. The difference was statistically significant apart from dyspnea, diarrhea and financial difficulties (see Table 4).

### Evolution according to treatment

**Fig. 1:**
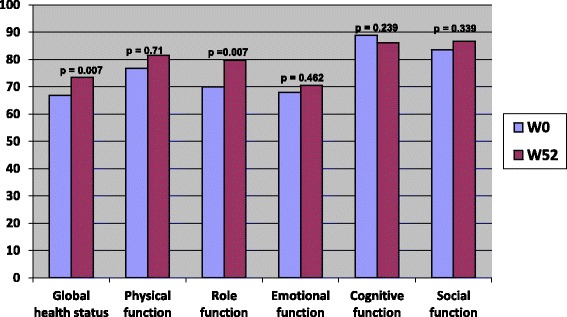
QLQ C30 functional scores before and after treatment by chemotherapy, among colorectal cancer patients, Morocco, 2009–2011

**Fig. 2:**
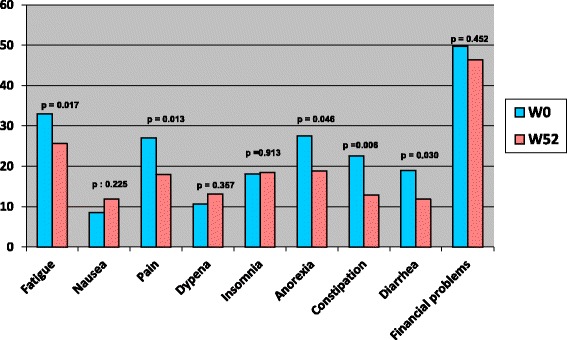
QLQ C30 symptoms scores before and after treatment by chemotherapy, among colorectal cancer patients, Morocco, 2009–2011

Overall, Functional dimensions scores were improved after the end of chemotherapy. However, this improvement was significant only for global health status and role functioning (66.93 vs 73.4 and 70 vs 79.8 respectively) (Fig. 1). A trend to an improvement was also seen for symptoms. The scores of fatigue, pain, appetite loss, constipation and diarrhea significantly decreased after completion of chemotherapy (Fig. 2).

**Fig. 3:**
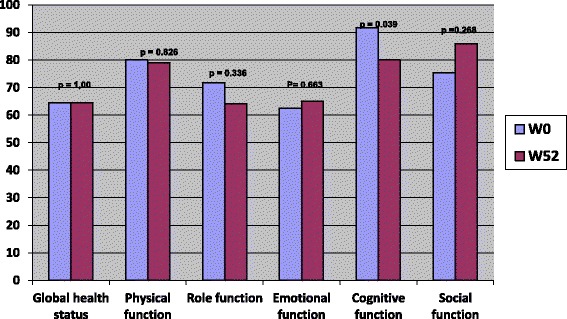
QLQ C30 functional scores before and after treatment by radiotherapy, among colorectal cancer patients, Morocco, 2009–2011

**Fig. 4:**
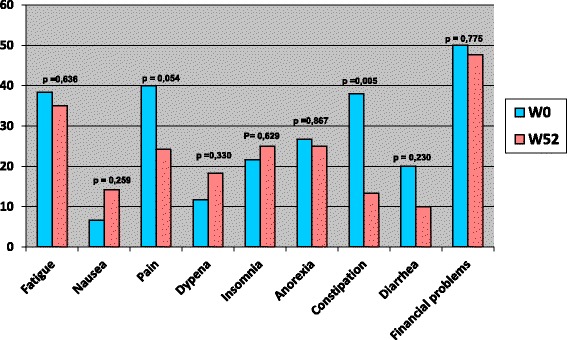
QLQ C30 symptoms scores before and after treatment by radiotherapy, among colorectal cancer patients, Morocco, 2009–2011

Curiously, the cognitive function was the only dimension which had a statistically significant variation. The cognitive capacities were altered after treatment by radiotherapy (91 vs 80 *p* = 0.04) (Fig. 3). Likewise, except for constipation, the symptoms scores did not differ significantly for patients treated by radiotherapy, between inclusion and at one year (Fig. 4). The constipation score fell from 38 to 13.33 (*p* = 0.005). Moreover, there was a borderline trend to an improvement in pain scores (40 vs 24.16 *p* = 0.054) (Fig. 4).

**Fig. 5:**
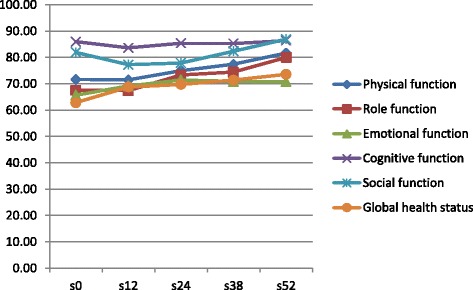
Evolution of functional dimensions every three months

**Fig. 6:**
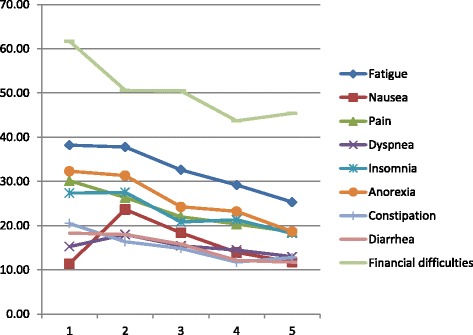
Evolution of symptoms every three months

When we look to the evolution of QOL scores every three months: overall, functional dimensions were stable at 12 weeks and have started to improve thereafter. The global health status began to improve after inclusion, whereas social function has slightly worsened at 12 weeks and increased thereafter (Fig. 5). Majority of symptoms were also stable at 12 weeks and decreased with time. Some symptoms have started to improve at 12 weeks like pain and constipation. Nausea was the only symptom that worsened at 12 weeks and improved thereafter (Fig. 6).

## Discussion

This is the first large prospective study assessing the quality of life of early colorectal cancer patients, in Morocco, and also in North Africa. The objective of this study was also to compare quality of life, before and after specific cancer treatment (chemotherapy and/or radiotherapy), at one year, when acute treatment effects are expected to have declined, to identify other factors which are related to this quality of life (age, sex, localization: colon or rectum, performance status, disease stage), to describe Moroccan cancer patients characteristics. In our study, the global health status was the most affected functional dimension at inclusion. For symptoms: financial difficulties and fatigue scores were the main factors affecting colorectal cancer patient’s Qol. The most important factors affecting Qol were: rectal location, advanced disease (stage III). The impact of age and gender on Qol was less important, mainly the impact of advanced age was equivocal.

The data on socio-demographic characteristics of the Moroccan population are important to describe, they may explain some Qol aspects. About one third of patients were less than 50 years old, the median age at diagnosis was comparable to the one found in a study assessing quality of life of Malaysian colorectal cancer patients [7] but it was lower than the European average [8]. There was also a slight male predominance, which is in agreement with other studies [7–9]. This study reports the socio-economic characteristics of Moroccan colorectal cancer patients; they are consistent with the results of previous studies examining psychosocial characteristics of Moroccan cancer patients [10, 11]. Most of patients are illiterate, have low income. However the rate of medical insurance is higher in this study (40 %) while it was not exceeding 15 % in previous Moroccan cohorts [10, 11], this could be explained by a new health policy of the Moroccan government. Moreover, majority of patients came from urban areas (78 %), in contradiction with previous studies where this rate was between 30 and 40 % [8, 9], and this could be explained, by the availability of new structures treating cancers in most of Moroccan regions.

It was interesting to assess the quality of life of Moroccan cancer patients in this particular context of poverty and illiteracy, while the health system, and in particular management of cancer is improving. Another proof of better fight against cancer is the diagnosis of colorectal cancer at a non-metastatic stage in more than 60 % of cases, while this percentage was previously found for metastatic disease. However most of patients had a stage III disease (66.3 %), which is comparable to the results of Malaysian and Chinese studies [7, 12]. In developed countries, earlier stages, I and II, are the most frequent [13]. This could be explained by the absence of a screening program in Morocco, in addition to the other socioeconomic characteristics of Moroccan patients. The proportion of rectal cancers (45 %) is high in this study, as it was reported in Moroccan registries, in comparison with European studies. In a French cohort, examining colorectal cancer quality of life, rectal cancer represented 36 % [1].

At inclusion, the global health status was comparable to an American population [9] and the EORTC reference values [8]. However, it was lower than a Malaysian population. (See Table 5).

**Table 5:** Comparison of QLQ-C30 scores at inclusion between Moroccan colorectal cancer patients and patients of three others populations

Item	Moroccan population	Malaysian population	American population	EORTC reference values
Global health status	62.8	79	62.6	60.7
Physical function	71	83	78.6	79.2
Role function	67	79	70.3	70.4
Emotional function	65.65	86.4	70.6	68.9
Cognitive function	86	92.2	79.7	85.2
Social function	81	88.2	68.4	76
Fatigue	38.2	16.6	38.8	34.7
Nauseas	11.3	4	13.4	7.3
Pain	30.15	17.2	29.3	24
Dyspnea	15.26	8.7	19.5	17.4
Insomnia	32.3	20.7	33.7	30.5
Appetite loss	32.6	18	25.2	19.1
Constipation	20.4	8.67	17.5	15.8
Diarrhea	18.3	10.3	15.4	16.6
Financial difficulties	61.7	26	32.5	13.6

Physical, role and emotional functions scores are lower than those of other populations but they remain close to the EORTC reference values [8]. All these parameters may be related, to the socioeconomic status of Moroccan patients.

The social function score is well above American and European ones [1, 8, 9]. This could be due to the Moroccan culture that promotes social and family relationships [10, 11].

The financial difficulties are, by far, more present in the Moroccan population. This is because of the low socioeconomic status and the lack of medical insurance. The lowest score are found in the European’s, whose health system is very well designed [1, 8].

The other symptoms scores are higher than those of the Malaysian population, in spite of the fact that the Malaysian study included stage IV colorectal cancer [7]. Moroccan scores are close to EORTC reference values except for nauseas, pain and appetite losses, suggesting a need for better supportive care for our patients.

Emotional and social functions were significantly worse in rectal cancer. Most symptoms were more present in rectal cancer. The difference was statistically significant for Nauseas, pain, insomnia, anorexia, constipation and diarrhea. At inclusion, global health status score was significantly worse in stage III. Anorexia was the only parameter which was significantly more important among colorectal female patients. A german study, didn’t find significant differences, between patients with colon and rectal cancer, neither differences between male nor female [13]. An Austrian study focused on the Qol impact of gender and found no gender-specific reaction to disease [14].

For Patients over 70 years-old, the difference was statistically significant for the physical function item which was lower. The influence of age on the impact of the disease is equivocal. Older people certainly have a higher morbidity associated with the disease. However, studies have shown that they better accept the deterioration of their health. Younger patients may be more impaired quality of life especially in relation to the psychological impact of the disease and some symptoms that are seen more significantly [13]. In our study, patients less than 50 years-old had higher scores of nauseas and insomnia.

Overall, Functional dimensions scores were improved after the end of chemotherapy. The symptoms scores did not differ significantly for patients treated by radiotherapy, between inclusion and at one year. When we look to the evolution of QOL scores every three months: overall, functional dimensions and symptoms were stable at 12 weeks and have started to improve thereafter, so even during treatments, as chemotherapy or radiotherapy, the QOL was maintained. Nausea was the only symptom that worsened at 12 weeks and improved thereafter, since it is the main symptom of which patients undergoing chemotherapy complain. This finding is consistent with a german study, where adjuvant chemotherapy didn’t alter long term’s quality of life of early colorectal cancer patients, at one year [13]. In the subgroup treated by adjuvant chemotherapy, a significant improvement was seen in the global health status, role function, fatigue, pain, anorexia, constipation and diarrhea [13]. Several studies showed that with actual management of adjuvant chemotherapy, the Qol can decrease during treatement, but in a non statistiscally significant manner. However patients quickly recover their quality of life scores before treatment within a few months after completion of therapy [15, 16].

In the radiotherapy subgroup, the only significant change for functional dimensions was observed in the cognitive function. Unfortunately, we could not find any reasonable explanation. Moreover, constipation was significantly and largely improved, according to EORTC criteria [17–19]. Pre-operative radiotherapy or chemoradiation didn’t impact quality of life of early colorectal cancer patients. The main limitation of this study is the lack of Qol assessment before surgery, mainly for colon cancer. QOL effects seen could be due to the surgery or the subsequent treatments. The strengths of the reported study are in related with its multicentre characteristic, including all Moroccan oncology centers and using a reliable tool of quality of life assessment.

## Conclusion

This study shows that Moroccan colorectal cancer patients are a young population. A screening program and more generalized medical insurance may help diagnosing colorectal cancer at an earlier stage. QLQ C30 scores are overall comparable to the EORTC QLQ C30 reference values except for some symptoms, which could be improved by more supportive care. Neither chemotherapy, nor radiotherapy worsened the long term quality of life of early colorectal cancer patients. The management of colorectal cancer, in addition to specific treatments, must take into account the patients quality of life, to overcome some insufficiencies.

## Abbreviations

EORTC: European organization for Research and treatment of Cancer
PS: Performance status
QOL: Quality of life
